# "Locked Out of Caregiving": A Case Study of Dementia Caregiving During the Covid-19 Pandemic

**DOI:** 10.1093/geroni/igab046.2948

**Published:** 2021-12-17

**Authors:** Kristie Wood, Marie-Anne Suizzo

**Affiliations:** 1 University of Texas, Austin, Austin, Texas, United States; 2 UT Austin, UT Austin, Texas, United States

## Abstract

It is unclear how ambiguous loss in dementia caregiving is impacted by conditions of the Covid-19 pandemic. Ambiguous loss describes situations in which closure is impossible and ambiguities within a family system ensue. Two situations of ambiguous loss exist. In the first type, one is psychologically absent, yet physically present, e.g. when one has dementia. In the second type, one is physically absent but psychologically present, e.g. moving to a nursing home. Ambiguous loss theory was applied to longitudinal interviews with an adult-child caregiver (age=52) of a mother with dementia, who resided in memory care during the Covid-19 pandemic. Theoretical analysis revealed both types of ambiguous loss were experienced in the dementia caregiving relationship. This was embedded within ambiguous loss type 2 due to the Covid-19 pandemic, e.g. social distancing and quarantine practices led to physical estrangement from others and ambiguity ensued about when, or if, estrangement would end before resulting in death. Further, the coping mechanisms defined in the ambiguous loss framework: restructuring identity, finding meaning, gaining mastery, increasing ambivalence capacity, reframing attachments, and gaining hope, were compromised due to overarching ambiguous loss attributed to the pandemic. Continued panic and frustration regarding lack of communication with and access to the memory care center instilled a sense of being “locked out of caregiving.” Findings suggest dementia caregivers may experience both types of ambiguous loss compounded during the Covid-19 pandemic, suspending grief and coping processes, and inciting poorly understood needs and challenges that must be better understood to support dementia caregivers.

